# Chronic Pelvic Pain and Sexual Dysfunction Among Females and Males Receiving Treatment for Opioid Use Disorder

**DOI:** 10.3389/fpain.2021.787559

**Published:** 2022-01-11

**Authors:** Geetika Reichmann, Anna Beth Parlier-Ahmad, Lori Beck, Bhushan Thakkar, Meryl Alappattu, Jeff Boissoneault, Caitlin E. Martin

**Affiliations:** ^1^School of Medicine, Virginia Commonwealth University, Richmond, VA, United States; ^2^Department of Psychology, Virginia Commonwealth University, Richmond, VA, United States; ^3^Department of Family Medicine and Population Health, Virginia Commonwealth University, Richmond, VA, United States; ^4^Department of Obstetrics and Gynecology, Virginia Commonwealth University, Richmond, VA, United States; ^5^Department of Physical Therapy, University of Florida, Gainesville, FL, United States; ^6^Department of Clinical and Health Psychology, University of Florida, Gainesville, FL, United States; ^7^Institute for Drug and Alcohol Studies, Virginia Commonwealth University, Richmond, VA, United States

**Keywords:** chronic pelvic pain, sex differences, opioid use disorder, sexual dysfunction, buprenorphine

## Abstract

**Introduction:** Chronic pain brings complexity to opioid use disorder (OUD). Psychosocial and neurobiological risks for Chronic Pelvic Pain (CPP) and OUD overlap. The primary objective of this exploratory study is to compare sex-specific prevalence of CPP and sexual dysfunction between individuals receiving buprenorphine for OUD and a comparison group receiving treatment for other chronic medical conditions (CMC).

**Methods:** Participants from an OUD treatment (*n* = 154) and primary care clinic (*n* = 109) completed a survey between July 2019 and February 2020 assessing reproductive and sexual health. Sex*-*stratified CPP and pain interference measures were adapted from the Brief Pain Inventory for females, and for males, the Brief Male Sexual Function Inventory and NIH Chronic Prostatitis Symptom Index. The Male and Female Sexual Function Index assessed sexual dysfunction. Prevalence of CPP and sexual dysfunction between groups were compared using Pearson χ2 and Fisher's Exact tests.

**Results:** Participants were 54.4% female and 75.0% Black with almost half having a psychiatric diagnosis. Among OUD females, the highest pain severity reported was for menstrual-related pain, and for OUD males, testicular pain. CPP most interfered with mood in OUD females vs. sleep and enjoyment of life in OUD males. There were no differences in prevalence for global sexual dysfunction with 91.6% of females and 84.2% of males screening positive across groups.

**Discussion/Implications:** CPP and sexual dysfunction are important components of wellness and may play a role in OUD recovery trajectories. The value of addressing CPP and sexual dysfunction in tailored comprehensive, sex-informed OUD treatment approaches should be further investigated.

## Introduction

Chronic pain is an important co-morbid condition that brings complexity to opioid use disorder (OUD) risk, course, and treatment (1, 2). The majority of patients receiving medication for opioid use disorder have at least one chronic pain condition, and for many patients, chronic pain was an antecedent medical condition leading to the development of their substance use disorder (1, 2).

Chronic Pelvic Pain (CPP), a specific subset of chronic pain, is an important yet underreported multifactorial condition for both sexes. The incidence of CPP is common with an estimated global prevalence of 5.7–26.6% in females (3) and 2–16% in males (4). The American College of Obstetrics and Gynecology defines CPP as “pain symptoms perceived to originate from pelvic organs/structures typically lasting more than 6 months” (5). Associated CPP symptoms in females may include pain with sexual intercourse, unexplained genital pain and/or itching, menstrual pain and pelvic cramping. In males, chronic prostatitis/chronic pelvic pain syndrome, also known as NIH Category III Prostatitis, is defined by the NIH as “the presence of genito-urinary pain in the absence of uropathogenic bacteria detected by standard microbiological methodology” (6). The main CPP domains in males include urogenital discomfort, sexual dysfunction, urinary tract symptoms (i.e., pain with voiding), and perineal pain. Given the clinical parameters of CPP, it is no surprise that CPP is also closely linked with sexual dysfunction in both males and females, impacting emotional status and overall quality of life (7).

CPP and substance use disorders share considerable psychosocial factors, such as PTSD history, trauma, sleep disorders, anxiety and depression (8, 9). CPP and substance use disorders also intersect neurobiologically, especially when considering neural pain-reward pathways (10). Given the overlapping factors contributing to both CPP and OUD, there is a predictable comorbidity between these two disorders, and their managements may complicate one another. For example, poorly controlled CPP may lead to instability among patients receiving medication treatment for OUD, potentially manifesting as increased opioid cravings and relapse risk. Existing data on substance use among CPP patients is limited, but when reported, suggests that substance use may be more prevalent among these patients (11, 12). However, to our knowledge, the prevalence of CPP within an OUD treatment population is unknown. Closing this gap in knowledge is an important first step to achieving a better understanding of how to manage patients with co-morbid CPP and OUD. Additionally, OUD and OUD medication treatment have been linked with sexual dysfunction, which may further complicate recovery and other health outcomes for these patients (13–15).

The development of personalized treatments for OUD, which include addressing co-morbid conditions and tailoring treatment components by sex and gender, is a high priority area urgently in need of more research and intervention in the current opioid crisis (16). Therefore, the primary objective of this exploratory study is to compare sex-specific prevalence of CPP and sexual dysfunction between individuals receiving buprenorphine for OUD and a comparison sample of patients receiving treatment for other chronic medical conditions. We chose to compare the prevalence of CPP and sexual dysfunction between addiction medicine and primary care populations as both are outpatient clinics focused on chronic disease management. This rationale allows our findings to align with the ongoing paradigm shift in addiction medicine where SUD treatments are best delivered within a chronic disease model vs. an acute care model (17). The overall purpose of this study is to generate hypotheses for further investigation; however, we did expect both CPP and sexual dysfunction to be highly prevalent in both samples.

## Methods

### Participants and Design

The current study involves a cross-sectional survey with medical record review of a convenience sample of patients recruited from an outpatient substance use disorder treatment center and a primary care clinic within the same academic medical center. Participants completed a voluntary, electronic survey assessing reproductive and sexual health needs. Study participants were English-speaking and at least 18 years old. All current patients at the addiction medicine clinic were eligible, whereas patients at the primary care clinic who were currently receiving substance use disorder treatment were not eligible. Participants who were unable to read had the option to have the survey read aloud by a research assistant in a private space (*n* = 6). Survey completion took an average of 40 minutes, and participants were compensated $20. A retrospective medical record chart abstraction was conducted for all participants. All participants provided verbal consent. This study was approved by the Institutional Review Board.

For this planned secondary data analytic study, we compared by sex participants from the two recruitment locations: the outpatient substance use disorder treatment center (OUD treatment group; *N* = 154; females: *n* = 84, males: *n* = 70) where data was collected from July through September 2019 and the primary care clinic [Chronic Medical Conditions (CMC); *N* = 109; females: *n* = 59, males: *n* = 50] where data was collected from December 2019 through February 2020. Participants who met the following criteria were included in analyses: (1) completion of the CPP survey measures and (2) completion of the sexual dysfunction survey measures. For participants in the OUD group, additional criteria included diagnosis of OUD and receiving buprenorphine at the time of the survey.

### Outpatient Substance Use Disorder Treatment Center

The outpatient substance use disorder treatment center study site opened in April 2017 and provides outpatient addiction treatment for over 500 adults, with approximately 90% receiving treatment for OUD with buprenorphine. The clinic is designated by state Medicaid services as a “preferred office-based opioid treatment center” and is affiliated with a large academic medical center with most patients referred by a provider within the academic medical center (e.g., inpatient consults, primary care physicians). Onsite addiction medicine providers come from multiple specialties, ranging from psychiatry to emergency medicine.

### Primary Care Clinic

Participants comprising the comparison group were recruited from an Internal Medicine clinic affiliated with the same academic medical center as the substance use disorder treatment clinic. This clinic serves as a safety net for the region and provides medical care to approximately 9,000 patients, age 18 and older, who account for more than 32,000 visits yearly.

### Demographic, Psychosocial and Clinical Characteristics

Demographic survey items included age, sex assigned at birth (male, female, other), gender, race, and annual income. Psychosocial survey variables included homelessness, sexual orientation, and education.

Clinical variables abstracted from the participants' medical records included insurance status, psychiatric and medical comorbidity diagnoses, and substance use history. Detailed substance use disorder treatment information (i.e., receiving buprenorphine for OUD) was abstracted from clinical intake assessments.

### Chronic Pelvic Pain Measures

#### Females

In females, pelvic pain was assessed through survey items adapted from existing CPP literature and clinical intake forms.

For females, having CPP was defined as those answering “yes” to the screening question “*During the past 6 months have you had pain or cramps in the pelvis and/or lower abdomen that has interfered with your daily activities?”* adapted from Lamvu et al. (18), and/or answering “yes” to any of the following vulvodynia screening questions over the past 6 months: “*Have you experienced itching in your genital area that persisted for 3 months or longer?,” “Have you experienced burning in your genital area that persisted for 3 months or longer?,” or “Have you experienced periodic knife-like or sharp pain in your genital area that persisted for 3 months or longer?”* adapted from Harlow et al. (19).

#### Males

In males, pelvic pain was evaluated via survey items modified from the Brief Male Sexual Function Inventory (20) and NIH Chronic Prostatitis Symptom Index (21).

For males, CPP is defined as answering “*Sometimes”* or more for at least one body part to the question “*How often have you experienced pain or discomfort in any of the following areas over the past 6 months?” (*answer options included: *Never, Rarely, Sometimes, Often, Usually, Always, and Not applicable): “Area between rectum and testicles (perineum),” “testicles,” “tip of the penis (not related to urination),” “below your waist,” “in your pubic or bladder area,” “pain or burning during urination,” “pain or discomfort during or after sexual climax (ejaculation),” “pain or discomfort as your bladder fills,” “pain or discomfort relieved by voiding”* (adapted from the NIH Chronic Prostatitis Symptom Index).

#### Males and Females

Additional pain indices were captured using adapted survey items from the Brief Pain Inventory (22) and existing clinical intake forms.

For *pain type and severity*, participants rated the severity of pain in the past 6 months on a scale of 0 (no pain) to 10 (worst pain imaginable) with urination, full bladder, and bowel movements. Additionally, females rated pain with deep intercourse, entry during intercourse, tampon insertion, other contact with genital area, pain the week before, during, and after menstrual period. Males rated pain in the area between rectum and testicles (perineum), testicles, at the tip of the penis (not related to urination), below waist in pubic or bladder area, pain or burning during urination, pain or discomfort during or after sexual climax (ejaculation).

For *pain intensity and interference*, participants rated their pelvic pain on a scale of 0 (no pain) to 10 (worst pain imaginable) for the following questions: “*Your pelvic pain at its worst in the last week,” “Your pelvic pain at its least in the last week,” “Your pelvic pain on average,” “Your pelvic pain right now.”* Pain interference was assessed by participants rating how much their pelvic pain has interfered with the following parameters in the past week (0 = does not interfere, 10 = completely interferes): general activity, mood, walking ability, normal activity (outside the home or with housework), relations with other people, sleep, enjoyment of life, sexual function (desire, interest, arousal, or satisfaction).

Lastly, to explore the relationship between pelvic pain and substance use, participants completed a novel survey item, “*When my pelvic pain is worse than usual, I am less or more likely to use drugs and/or alcohol,”* and “*My use of drugs and/or alcohol relieves my pelvic pain,”* and responded on a visual analog scale (range 0–100 with 50 indicating not more or less likely).

### Sexual Dysfunction Measures

#### Females

The abbreviated 6-item Female Sexual Function Index (FSFI) by Isidori et al. (23) evaluated sexual dysfunction (1–5 Likert scale). The FSFI is a validated tool used to assess six domains of female sexual function including desire, arousal, lubrication, orgasm, satisfaction and pain. An item score of <4 indicates dysfunction within a domain. A total score of <19 indicates sexual dysfunction (total score range: 6–30).

#### Males

Sexual dysfunction was assessed with the 11-item Brief Sexual Function Inventory (BSFI; 0–4 Likert scale). The BSFI is a validated questionnaire that assess three domains of male sexual function including sexual drive (2 items), erectile function (3 items) and ejaculatory function (2 items), as well as problem assessment (3 items) and overall satisfaction (1 item). Based on literature by Dahl et al. (24), cutoffs for dysfunction within each domain are as follows: drive ≤3, erection ≤7, ejaculation ≤5, and satisfaction ≤ 1. A combined score for drive, erection, and ejaculation (DEE) was also created, and defined a problem in this domain as DEE ≤ 10. Overall sexual dysfunction was defined as the presence of either a satisfaction problem or a DEE problem (24).

#### Analysis

For the primary objective, sex stratified CPP and sexual dysfunction prevalence and descriptors were compared between the OUD and CMC groups using Pearson χ2 and Fisher's Exact tests for categorical variables and *T*-tests for continuous variables. For all analyses, significance was set at 0.05. Statistical analyses were performed using SAS 9.4 (SAS Institute Inc., Cary, NC).

## Results

### Demographic and Psychosocial Characteristics

A total of 263 participants (97% response rate) were included in the analyses with 84 females and 70 males in the OUD group and 59 females and 50 males in the CMC group. All participants meeting inclusion criteria for this study identified as cisgender. Demographic and psychosocial characteristics are presented in Table 1. Most participants self-identified as Black (75%) with a mean age of 49.4 (±13.3) years. No individuals self-identified as Latinx ethnicity. Compared to CMC participants, males and females in the OUD group were significantly more likely to be homeless (OUD females: 30.5%, CMC females: 12.7%, *p* = 0.016; OUD males: 38.6%, CMC males: 12.2%, *p* = 0.002), and have psychiatric comorbidities such as PTSD (OUD females: 13.4%, CMC females: 2.2%, *p* = 0.046; OUD males: 16.2%, CMC males: 2.0%, *p* = 0.014). Medical comorbidities were more common in CMC than OUD participants (OUD females: 80.9%, CMC females: 97.8%, *p* = 0.008; OUD males: 72.9%, CMC males: 98.0%, *p* = <0.001). Almost half of all participants reported having a co-morbid pain condition other than CPP.

**Table 1:** Demographic and psychosocial characteristics of study sample.

**Study sample characteristics**	**Females** ***N*** **= 143**			**Males** ***N*** **= 120**		
	**OUD group *N* = 84 (%)**	**CMC group *N* = 59 (%)**	***P* ^b^**	**OUD group *N* = 70 (%)**	**CMC group *N* = 50 (%)**	***P* ^b^**
**Age (Mean ± SD)**	39.9 ± 11.6	57.8 ± 9.9	**<0.0001**	46.9 ± 12.4	58.4 ± 7.7	**<0.0001**
**Self-identified race**			**<0.0001**			**0.044**
Black	54 (65.1)	50 (84.8)		52 (75.4)	40 (81.6)	
White	25 (30.1)	3 (5.1)		12 (17.4)	7 (14.3)	
Other	4 (4.8)	6 (10.2)		5 (7.3)	2 (4.1)	
**Sexual orientation**			**0.0004**			**0.047**
Heterosexual	59 (70.2)	50 (90.9)		64 (91.4)	43 (87.8)	
Gay or lesbian	8 (9.5)	0 (0.0)		1 (1.4)	3 (6.1)	
Bisexual/other	17 (20.2)	5 (9.1)		5 (7.1)	3 (6.1)	
**Education**			0.782			**0.029**
< High school degree	15 (17.9)	12 (20.3)		18 (25.7)	11 (22.5)	
High school degree/equivalent	42 (50.0)	26 (44.1)		35 (50.0)	15 (30.6)	
>High school degree	27 (32.1)	21 (35.6)		17 (24.29)	23 (46.9)	
**Annual household income**			0.051			0.117
< $5000	41 (50.6)	17 (30.4)		32 (47.1)	14 (28.6)	
$5,000–$10,000	16 (19.8)	13 (23.2)		11 (16.2)	9 (18.4)	
>$10,000	24 (29.6)	26 (46.4)		25 (36.8)	26 (53.1)	
**Homeless**	25 (30.5)	7 (12.7)	**0.016**	27 (38.6)	6 (12.2)	**0.002**
**Psychiatric comorbidity**						
Depression	30 (44.8)	17 (37.0)	0.407	36 (53.7)	14 (28.6)	**0.007**
Anxiety/Panic disorder	20 (29.9)	7 (15.2)	0.073	23 (34.3)	7 (14.3)	**0.015**
PTSD	9 (13.4)	1 (2.2)	**0.046**	11 (16.2)	1 (2.0)	**0.014**
Bipolar disorder	9 (13.4)	2 (4.4)	0.195	9 (13.4)	1 (2.0)	**0.043**
**Co-morbid conditions**						
Medical comorbidity	55 (80.9)	45 (97.8)	**0.008**	51 (72.9)	48 (98.0)	**<0.001**
Pain comorbidity (non-CPP pain)	36 (42.9)	41 (69.5)	**0.002**	31 (44.3)	25 (50.0)	0.536
Chronic pelvic pain (Y/N)^a^	17 (22.7)	11 (22.0)	0.930	24 (34.3)	10 (20.0)	0.103

*OUD, Opioid Use Disorder; CMC, Chronic Medical Conditions*.

a *For males, CPP is defined as answering “Sometimes” for at least one body part to the question “How often have you experienced pain or discomfort in any of the following areas over the past 6 months?” For females, having CPP is defined as those answering “yes” to the CPP screening question “During the past 6 months have you had pain or cramps in the pelvis and/or lower abdomen that has interfered with your daily activities” and/or answering “yes” to any of the vulvodynia screening questions over the past 6 months (see methods for full screening questions)*.

b *Boldface indicates significance at p < 0.05*.

#### Chronic Pelvic Pain

Table 2 describes the prevalence of CPP characteristics between the OUD and CMC groups. Less than a quarter of females (*n* = 28; 19.6%) and almost a third of males (*n* = 34; 28.3%) met clinical criteria for CPP with no significant differences in prevalence between OUD and CMC groups. However, females and males with CPP in the OUD group rated their average CPP intensity higher (albeit not statistically significant) than females and males in the CMC group (OUD females: 3.0 ± 2.9, CMC females: 0.6 ± 1.0, *p* = 0.189; OUD males: 3.6 ± 3.0, CMC males: 1.9 ± 2.3, *p* = 0.151). Regarding pain severity indices, OUD females with CPP reported menstrual pain to be the most severe and reported higher pain severity indices than CMC females with full bladder, bowel movements and tampon insertion. OUD males with CPP reported significantly higher testicular pain severity (OUD males: 5.1 ± 3.1, CMC males: 2.1 ± 2.1, *p* = 0.017) than CMC males as well as higher pain severity with perineal pain, pain at the tip of the penis, pubic/bladder pain, pain/burning with urination and pain during/after ejaculation. For pain interference domains, OUD females reported that their CPP displayed the highest pain interference with their mood, normal household activity and sexual function. OUD males reported the highest CPP pain interreference with their sleep, enjoyment of life and sexual function (Table 2).

**Table 2:** Pain parameters, severity indices, and interference indices among participants with CPP.

**CPP parameters**	**Females**			**Males**		
	**OUD + CPP (*n* = 17)**	**CMC + CPP (*n* = 11)**	***P* ^d^**	**OUD + CPP (*n* = 24)**	**CMC + CPP (*n* = 10)**	***P* ^d^**
**Average pain intensity (range 0–10 ± SD)**	3.0 ± 2.9	0.6 ± 1.0	0.189	3.6 ± 3.0	1.9 ± 2.3	0.151
**Pain severity indices (range 0–10 ± SD)**						
Pain with urination	1.5 ± 1.9	2.5 ± 3.0	0.763	–	–	–
Pain with full bladder	3.0 ± 3.6	1.6 ± 2.3	0.188	–	–	–
Pain with bowel movements	3.4 ± 3.7	1.8 ± 2.8	0.310	2.3 ± 3.0	1.5 ± 2.2	0.310
Deep pain with intercourse	2.0 ± 2.6	2.3 ± 3.1	0.636	–	–	–
Pain with tampon insertion	2.2 ± 3.2^a^	0.5 ± 0.1^b^	1.00	–	–	–
Pain with genital contact	1.2 ± 1.7	1.5 ± 2.3	0.919	–	–	–
Pre-menstrual pain	2.0 ± 3.7^a^	0.9 ± 4.3^b^	0.495	–	–	–
Menstrual pain	5.8 ± 3.2^a^	0.8 ± 7.1^b^	0.182	–	–	–
Post-menstrual pain	2.7 ± 3.6^a^	3.1 ± 2.8^b^	0.505	–	–	–
Perineal pain	–	–	–	4.5 ± 3.5	2.4 ± 2.8	0.137
Testicular pain	–	–	–	5.1 ± 3.1	2.1 ± 2.1	**0.017**
Pain at tip of penis (unrelated to urination)	–	–	–	5.0 ± 3.5	1.8 ± 1.3	0.182
Pubic/bladder pain	–	–	–	5.0 ± 3.1	3.3 ± 3.3	0.143
Pain/burning during urination	–	–	–	4.3 ± 3.0	3.0 ± 3.7	0.321
Pain during/after ejaculation	–	–	–	4.8 ± 3.6	2.2 ± 2.2	0.182
**Pain interference indices (range 0–10 ± SD)**						
General activity	1.9 ± 3.5	1.7 ± 2.9^c^	0.590	3.6 ± 3.6	1.8 ± 2.5	0.076
Mood	4.6 ± 4.6	1.7 ± 2.8^c^	0.431	3.5 ± 3.4	2.9 ± 3.2	0.499
Walking ability	2.9 ± 35.4	1.7 ± 2.9^c^	0.419	3.8 ± 3.6	3.9 ± 3.9	0.885
Normal household activity	3.6 ± 3.7	1.7 ± 2.9^c^	0.419	3.8 ± 3.4	3.9 ± 3.7	0.988
Relations with other people	1.7 ± 2.7	1.7 ± 2.9^c^	0.549	3.2 ± 3.3	2.0 ± 2.5	0.251
Sleep	2.8 ± 3.1	2.4 ± 4.2^c^	0.874	4.6 ± 3.3	3.2 ± 3.4	0.154
Enjoyment of life	2.9 ± 2.9	0.0 ± 0.0^c^	0.120	4.7 ± 3.7	3.0 ± 2.9	0.104
Sexual function (desire, interest, arousal or satisfaction)	3.4 ± 4.8	1.7 ± 2.9^c^	0.608	4.4 ± 3.4	3.2 ± 3.7	0.226

*OUD, Opioid Use Disorder; CMC, Chronic Medical Conditions; CPP, Chronic Pelvic Pain*.

a *For these parameters, n = 6*;

b *For these parameters, n = 2*;

c *For these parameters, n = 3*;

d *Boldface indicates significance at p < 0.05*.

With the exploratory item (data not shown), OUD males were significantly more likely than CMC males to report using drugs/alcohol during CPP exacerbations (OUD males: 54.1 ± 34.0, CMC males: 19.9 ± 31.1, *p* = 0.003) and that the use of drugs/alcohol relieved their CPP (OUD males: 50.2 ± 31.8, CMC males: 18.1 ± 30.0, *p* = 0.002). No significant differences existed for females; however, OUD females reported higher likelihood of using drugs/alcohol during CPP exacerbations (OUD females: 52.2 ± 41.1, CMC females: 16.7 ± 28.7, *p* = 0.417) and that drugs/alcohol relieved their CPP (OUD females: 26.0 ± 35.9, CMC females: 17.0 ± 28.6, *p* = 0.892) than females in the CMC group.

### Sexual Dysfunction

Table 3 describes the prevalence of sexual dysfunction and its characteristics for the OUD and CMC groups. Overall, 91.6% of females and 84.2% of males screened positive for global sexual dysfunction with no significant differences in prevalence between OUD and CMC groups. More males and females within the CMC group met criteria for dysfunction in each of the individual sexual dysfunction domains as compared to their respective OUD groups. Notably, CMC females were significantly more likely than OUD females to show dysfunction in the domains of arousal (*p* = 0.028), lubrication (*p* = 0.004), and orgasm (*p* = 0.042).

**Table 3:** Sexual dysfunction characteristics among males and females in the study sample.

**Sexual dysfunction domains**	**Females** ***N*** **= 143**			**Males** ***N*** **= 120**		
	**OUD group *N* = 84 (%)**	**CMC group *N* = 59 (%)**	***P* ^f^**	**OUD group *N* = 70 (%)**	**CMC group *N* = 50 (%)**	***P* ^f^**
**Global sexual dysfunction (Y/** ***N*** **)** ^**a,b**^	78 (92.9)	53 (89.8)	0.521	58 (89.2)	43 (91.5)	0.759
**Female sexual dysfunction domains** ^**c**^						
Desire	78 (100.0)^e^	52 (100.0)^e^	1.000	–	–	–
Arousal	58 (75.3)	48 (90.6)	**0.028**	–	–	–
Lubrication	48 (61.5)	45 (84.9)	**0.004**	–	–	–
Orgasm	53 (68.8)	44 (84.6)	**0.042**	–	–	–
Penetrative pain	34 (43.6)	24 (46.2)	0.773	–	–	–
Sexual satisfaction	29 (37.2)	22 (43.1)	0.499	–	–	
**Male sexual dysfunction domains** ^**d**^						
Drive	–	–	–	32 (45.7)	26 (52.0)	0.497
Erection	–	–	–	36 (51.4)	30 (60.0)	0.352
Ejaculation	–	–	–	44 (62.9)	37 (74.0)	0.199

*OUD, Opioid Use Disorder; CMC, Chronic Medical Conditions*.

a *In females, global sexual dysfunction was defined as a total score of <19 (range: 6–30) on the abbreviated FSFI*;

b *In males, global sexual dysfunction was defined as the presence of either a satisfaction problem or a DEE (drive, erection, and ejaculation) problem based on the BSFI and literature adapted from Dahl et al.*;

c *Data reflect proportion of participants screening positive for dysfunction in domain by an item score of <4*;

d *Data reflect proportion of participants screening positive for dysfunction in domains: drive (score <3), erection (<5), and ejaculation (<7)*;

e *13 females skipped this question*;

f *Boldface indicates significance at p <0.05*.

## Discussion

CPP is a prevalent and significant chronic pain disorder seen in both males and females. The purpose of this secondary analytic study was to describe the sex-specific prevalence of CPP and sexual dysfunction in patients receiving buprenorphine for OUD compared to patients being treated for other chronic conditions. Overall, our study results demonstrate that 1 in 5 females and over 1 in 4 males met clinical criteria for CPP. We found no difference in CPP prevalence between the two groups, but the OUD group tended to report slightly greater pain severity in our small sample, a finding that should be investigated further in larger samples. OUD males with CPP were significantly more likely to report using drugs/alcohol during CPP exacerbations; no such significant differences existed for females with co-morbid OUD and CPP. Regarding sexual dysfunction, the majority of females and males screened positive, but there were no differences in global sexual dysfunction prevalence between groups; however, a greater proportion of males and females within the CMC group met criteria for dysfunction within each domain than those in the OUD group.

CPP is an important component of health and wellness for both sexes and thus may also play a role in the recovery trajectories for individuals with OUD. In our sample, participants reported that CPP interfered with aspects of life that also impact OUD treatment outcomes, such as mood, sleep, and enjoyment of life. Females with OUD and CPP reported that their pain interfered the most with their mood. Several studies have documented that negative affect and pain may serve as triggers for sustained opioid use and substance use recurrence for individuals with OUD (25, 26). Thus, our study findings highlight the potential role CPP may be playing in the intersection between mood, pain, and addiction, especially among female patients receiving medication treatment for OUD. Further research using sex-stratified longitudinal data is needed to discern chronology of how CPP, other pain conditions, depression, and anxiety symptoms can lead to comprised OUD treatment outcomes. Additionally, menstrual pain appeared to be the most severe pain reported by OUD females with CPP, a type of pain with available treatments. However, treatments are only effective if they are provided and utilized, underlying the need for more evidence to guide best practices to integrate reproductive and sexual health care into OUD treatment (27).

Males with OUD reported the highest CPP interference with their sleep and overall enjoyment of life. Sleep and quality of life are intertwined, and addressing sleep disturbances is an avenue to improve health outcomes (28). Opioids are known to precipitate sleep disturbances, and sleep interruptions may in turn increase susceptibility for opioid misuse (29). Further, emerging data suggests a relationship between poor sleep and disruption of the brain reward system, which may facilitate persistent opioid use (30). Considering individuals in OUD treatment, sleep is increasingly being recognized as a potential target to improve outcomes, such as via relapse prevention (31). Future research investigating avenues to integrate sleep health interventions into medication treatment for OUD should prioritize simultaneously measuring chronic pain indices, such as pelvic pain, and analyzing pain as a potential mediator of treatment response.

Another interesting finding in this study was the connection between CPP exacerbations and self-reported risk of subsequent drug/alcohol use by OUD males and females. This exploratory finding suggests that CPP may impact the risk of substance use even after addiction treatment engagement, and CPP may therefore compromise treatment outcomes. The neurobiological mechanisms underlying CPP are complex. It is theorized that repetitive visceral and somatic pain inputs from malfunctioning pelvic structures to the spinal cord may result in hypersensitivity to pain, decreased pain inhibition, and an overall increased sensitization of the central nervous system to pain (7, 32). This heightened response to pain and its management is especially challenging when considering an OUD population. The experience of chronic pain during OUD treatment facilitates opioid cravings (33) and is a predictor for subsequent non-prescribed opioid use recurrence (34). Given these potential implications, further research analyzing the relationship that chronic pain disorders, such as CPP, have with OUD course and treatment in both sexes is warranted.

In this study, most participants screened positive for sexual dysfunction, with greater proportion of CMC participants demonstrating higher prevalence of dysfunction in individual domains. The pathogenesis of sexual dysfunction is multi-factorial and complex (35, 36). Our finding of such a high prevalence of sexual dysfunction across both groups may be driven by the substantial co-morbid psychiatric, medical, and psychosocial burden on our OUD and CMC participants (37–39). For our CMC participants, there is need for more research and scientific reporting on prevalence, pathophysiology, and optimal treatment of sexual dysfunction associated with chronic illness; additionally, screening for and managing co-morbid psychiatric conditions like depression is recommended for patients with sexual dyssfunction. For OUD participants, these risk factors are complicated further by how opioids disrupt the hypothalamic–pituitary–gonadal axis, resulting in hypogonadism and impaired sexual function (13). CPP also impacts sexual function, with cohort studies reporting erectile dysfunction in 15–55% of males with CPP (40) and meta-analyses demonstrating lower FSFI scores in lubrication, pain, and total score domains in females with CPP compared to controls (41). Conversely, the effect of buprenorphine treatment on sexual dysfunction has not been well-studied. In a previous study by Ramdurg et al. approximately 83.0% of men receiving buprenorphine reported sexual dysfunction in at least one domain (14). A recent multicenter study reported that 56.6% of women receiving medication for OUD met criteria for sexual dysfunction (15). This common and unfavorable experience of sexual dysfunction during OUD treatment may influence an individual's decision to continue these lifesaving medications for OUD. Our findings reinforce the need for further investigation of how sexual dysfunction intersects with treatment and recovery among individuals receiving buprenorphine for OUD treatment.

### Limitations

This study has several limitations. Our study was conducted at two outpatient clinics within a single academic medical institution, so results may not generalize to other clinics or regions. Additionally, these findings may not generalize to individuals who are engaged with alternative treatment modalities or non-treatment seeking for OUD. The sample size of this study was small, possibly impacting the ability to detect significant results. Because both study sites recruited convenience samples, the OUD and CMC groups were not matched on sociodemographic characteristics such as age (i.e., older average age of CMC participants), possibly introducing confounders into analyses. Two different definitions for CPP were used for males and females which precluded our ability to compare prevalence between sexes. Assessment of CPP in both males and females was performed using survey items from different questionnaires due to lack of universally accepted measures for CPP, likely introducing bias into our results. Lastly, social desirability bias may have skewed our results as the survey was completely by self-report and asked about several sensitive issues, especially for the six participants for whom all the questions were read aloud by a research assistant. This study does not endorse changes to current OUD treatment or clinical practice guidelines; rather, this study is exploratory only and is meant to inform future work.

## Conclusions

To our knowledge, our study is the first to report on CPP and sexual dysfunction among a sample receiving buprenorphine for OUD treatment. Our study was further strengthened by including both sexes and the inclusion of a comparison group without OUD within the same academic medical center. CPP, and its effects on sexual dysfunction, is not currently assessed routinely in OUD treatment, which may represent a missed opportunity to strengthen recovery outcomes for patients with co-morbid CPP and OUD. These findings are meant to inform and support future work regarding the integration of sexual health and wellness into OUD treatment programs.

## Data Availability Statement

The raw data supporting the conclusions of this article will be made available by the authors, without undue reservation.

## Ethics Statement

The studies involving human participants were reviewed and approved by Virginia Commonwealth University Institutional Review Board. Written informed consent for participation was not required for this study and verbal consent was obtained in accordance with the institutional requirements.

## Author Contributions

CM, MA, and JB designed the study. AP-A, BT, and LB contributed to data collection. CM, AP-A, LB, and GR conceived the analysis design. LB performed the statistical analysis. AP-A, BT, GR, and CM contributed to data interpretation. GR wrote the initial draft. All authors contributed to the drafting of the manuscript, creating the final version, and authors approve the submitted version.

## Funding

This study was supported by the Jeanann Gray Dunlap Foundation and the National Center for Advancing Translational Sciences (UL1TR002649). CM is supported by NIDA K23 DA053507 from the National Institute on Drug Abuse. AP-A receives funding from the NIDA T32DA007027 award (PI: Dr. William Dewey). Funders were not directly involved in the preparation of this research manuscript. The authors alone are responsible for the content and writing of the paper.

## Conflict of Interest

The authors declare that the research was conducted in the absence of any commercial or financial relationships that could be construed as a potential conflict of interest.

## Publisher's Note

All claims expressed in this article are solely those of the authors and do not necessarily represent those of their affiliated organizations, or those of the publisher, the editors and the reviewers. Any product that may be evaluated in this article, or claim that may be made by its manufacturer, is not guaranteed or endorsed by the publisher.

## Appendix

**Table A1:** Age and sex specific prevalence of Chronic Pelvic Pain and Sexual Dysfunction amongst females and males in the CMC and OUD groups.

**Sample characteristics**	**Females**				**Males**			
**Age groups**	**Chronic pelvic pain**		**Sexual dysfunction**		**Chronic pelvic pain**		**Sexual dysfunction**	
	**CMC**	**OUD**	**CMC**	**OUD**	**CMC**	**OUD**	**CMC**	**OUD**
18–34	NA (0/0)	27% (8/30)	50% (0/1)	84% (31/37)	NA (0/0)	6% (2/17)	NA (0/0)	88% (14/16)
35–44	25% (1/4)	29% (4/14)	100% (4/4)	100% (16/16)	0 (0/2)	30% (3/10)	100% (2/2)	78% (7/9)
45–54	15% (2/13)	21% (4/19)	100% (14/14)	100% (19/19)	18% (2/11)	33% (6/18)	91% (10/11)	89% (16/18)
55–64	32% (7/22)	9% (1/11)	89% (24/27)	100% (11/11)	26% (6/23)	50% (11/22)	90% (18/20)	95% (18/19)
65+	9% (1/11)	50% (0/1)	85% (11/13)	100% (1/1)	14% (2/14)	67% (2/3)	93% (13/14)	100% (3/3)

*OUD, Opioid Use Disorder; CMC, Chronic Medical Conditions*.

## References

[1] Hser YI Mooney LJ Saxon AJ Miotto K Bell DS Huang D. Chronic pain among patients with opioid use disorder: results from electronic health records data. J Subst Abuse Treat. (2017) 77:26–30. 10.1016/j.jsat.2017.03.006 28476267 PMC5424616

[2] Speed TJ Parekh V Coe W Antoine D. Comorbid chronic pain and opioid use disorder: literature review and potential treatment innovations. Int Rev Psychiatry. (2018) 30:136–46. 10.1080/09540261.2018.1514369 30398071

[3] Ahangari A. Prevalence of chronic pelvic pain among women: an updated review. Pain Physician. (2014) 17:E141–7. 10.36076/ppj.2014/17/E141 24658485

[4] Smith CP. Male chronic pelvic pain: an update. Indian J Urol. (2016) 32:34–9. 10.4103/0970-1591.173105 26941492 PMC4756547

[5] Chronic pelvic pain: ACOG practice bulletin Number 218. Obstet Gynecol. (2020) 135:e98–109. 10.1097/AOG.0000000000003716 32080051

[6] Nickel JC Alexander RB Anderson R Berger R Comiter CV Datta NS. Category III chronic prostatitis/chronic pelvic pain syndrome: insights from the national institutes of health chronic prostatitis collaborative research network studies. Curr Urol Rep. (2008) 9:320–7. 10.1007/s11934-008-0055-7 18765132 PMC2829842

[7] Lamvu G Carrillo J Ouyang C Rapkin A. Chronic pelvic pain in women: a review. JAMA. (2021) 325:2381–91. 10.1001/jama.2021.2631 34128995

[8] Bilevicius E Sommer JL Asmundson GJG El-Gabalawy R. Posttraumatic stress disorder and chronic pain are associated with opioid use disorder: results from a 2012-2013 American nationally representative survey. Drug Alcohol Depend. (2018) 188:119–25. 10.1016/j.drugalcdep.2018.04.005 29775955

[9] Ross S Peselow E. Co-occurring psychotic and addictive disorders: neurobiology and diagnosis. Clin Neuropharmacol. (2012) 35:235–43. 10.1097/WNF.0b013e318261e193 22986797

[10] Leknes S Tracey I. A common neurobiology for pain and pleasure. Nat Rev Neurosci. (2008) 9:314–20. 10.1038/nrn2333 18354400

[11] Walker E Katon W Harrop-Griffiths J Holm L Russo J Hickok LR. Relationship of chronic pelvic pain to psychiatric diagnoses and childhood sexual abuse. Am J Psychiatry. (1988) 145:75–80. 10.1176/ajp.145.1.75 3337296

[12] Lamvu G Soliman AM Manthena SR Gordon K Knight J Taylor HS. Patterns of prescription opioid use in women with endometriosis: evaluating prolonged use, daily dose, and concomitant use with benzodiazepines. Obstet Gynecol. (2019) 133:1120–30. 10.1097/AOG.0000000000003267 31135725 PMC6553518

[13] Ortman HA Siegel JA. The effect of methadone on the hypothalamic pituitary gonadal axis and sexual function: a systematic review. Drug Alcohol Depend. (2020) 207:107823. 10.1016/j.drugalcdep.2019.107823 31901578

[14] Ramdurg S Ambekar A Lal R. Co-relationship between sexual dysfunction and high-risk sexual behavior in patients receiving buprenorphine and naltrexone maintenance therapy for opioid dependence. Ind Psychiatry J. (2015) 24:29–34. 10.4103/0972-6748.160930 26257480 PMC4525428

[15] Zamboni L Franceschini A Portoghese I Morbioli L Lugoboni F GICS Group. Sexual functioning and opioid maintenance treatment in women. results from a large multicentre study. Front Behav Neurosci. (2019) 13:97. 10.3389/fnbeh.2019.00097 31156404 PMC6528617

[16] Volkow ND. Personalizing the treatment of substance use disorders. Am J Psychiatry. (2020) 177:113–6. 10.1176/appi.ajp.2019.19121284 32008390

[17] Kelly JF White WL. Recovery management and the future of addiction treatment and recovery in the USA In: Kelly J White W, editors. Addiction Recovery Management: Current Clinical Psychiatry. New York, NY: Springer (2010). p. 303–316. 10.1007/978-1-60327-960-4_16

[18] Lamvu G Williams R Zolnoun D Wechter ME Shortliffe A Fulton G. Long-term outcomes after surgical and nonsurgical management of chronic pelvic pain: one year after evaluation in a pelvic pain specialty clinic. Am J Obstet Gynecol. (2006) 195:591–8; discussion 8–600. 10.1016/j.ajog.2006.03.081 16729951

[19] Harlow BL Vazquez G MacLehose RF Erickson DJ Oakes JM Duval SJ. Self-reported vulvar pain characteristics and their association with clinically confirmed vestibulodynia. J Womens Health. (2009) 18:1333–40. 10.1089/jwh.2007.1032 19743906 PMC2825727

[20] O'Leary MP Fowler FJ Lenderking WR Barber B Sagnier PP Guess HA. A brief male sexual function inventory for urology. Urology. (1995) 46:697–706. 10.1016/S0090-4295(99)80304-5 7495124

[21] Litwin MS McNaughton-Collins M Fowler FJ Jr Nickel JC Calhoun EA Pontari MA. The national institutes of health chronic prostatitis symptom index: development and validation of a new outcome measure. Chronic prostatitis collaborative research network. J Urol. (1999) 162:369–75. 10.1097/00005392-199908000-00022 10411041

[22] Cleeland CS Ryan KM. Pain assessment: global use of the Brief pain inventory. Ann Acad Med Singap. (1994) 23:129–38. 8080219

[23] Isidori AM Pozza C Esposito K Giugliano D Morano S Vignozzi L. Development and validation of a 6-item version of the female sexual function index (FSFI) as a diagnostic tool for female sexual dysfunction. J Sex Med. (2010) 7:1139–46. 10.1111/j.1743-6109.2009.01635.x 19968774

[24] Dahl AA Bremnes R Dahl O Klepp O Wist E Fosså SD. Is the sexual function compromised in long-term testicular cancer survivors? Eur Urol. (2007) 52:1438–47. 10.1016/j.eururo.2007.02.046 17350159

[25] McHugh RK Devito EE Dodd D Carroll KM Potter JS Greenfield SF. Gender differences in a clinical trial for prescription opioid dependence. J Subst Abuse Treat. (2013) 45:38–43. 10.1016/j.jsat.2012.12.007 23313145 PMC3626739

[26] Back SE Lawson KM Singleton LM Brady KT. Characteristics and correlates of men and women with prescription opioid dependence. Addict Behav. (2011) 36:829–34. 10.1016/j.addbeh.2011.03.013 21514061 PMC3164361

[27] MacAfee LK Harfmann RF Cannon LM Kolenic G Kusunoki Y Terplan M. Sexual and reproductive health characteristics of women in substance use treatment in michigan. Obstet Gynecol. (2020) 135:361–9. 10.1097/AOG.0000000000003666 31923070

[28] Espie CA Emsley R Kyle SD Gordon C Drake CL Siriwardena AN. Effect of digital cognitive behavioral therapy for insomnia on health, psychological well-being, and sleep-related quality of life: a randomized clinical trial. JAMA Psychiatry. (2019) 76:21–30. 10.1001/jamapsychiatry.2018.2745 30264137 PMC6583463

[29] Brower KJ Perron BE. Sleep disturbance as a universal risk factor for relapse in addictions to psychoactive substances. Med Hypotheses. (2010) 74:928–33. 10.1016/j.mehy.2009.10.020 19910125 PMC2850945

[30] Fathi HR Yoonessi A Khatibi A Rezaeitalab F Rezaei-Ardani A. Crosstalk between sleep disturbance and opioid use disorder: a narrative review. Addict Health. (2020) 12:140–58. 10.22122/ahj.v12i2.249 32782736 PMC7395935

[31] Greenwald MK Moses TEH Roehrs TA. At the intersection of sleep deficiency and opioid use: mechanisms and therapeutic opportunities. Transl Res. (2021) 234:58–73. 10.1016/j.trsl.2021.03.006 33711513 PMC8217216

[32] Aredo JV Heyrana KJ Karp BI Shah JP Stratton P. Relating chronic pelvic pain and endometriosis to signs of sensitization and myofascial pain and dysfunction. Semin Reprod Med. (2017) 35:88–97. 10.1055/s-0036-1597123 28049214 PMC5585080

[33] Tsui JI Lira MC Cheng DM. Chronic pain, craving, and illicit opioid use among patients receiving opioid agonist therapy. Drug Alcohol Depend. (2016) 166:26–31. 10.1016/j.drugalcdep.2016.06.024 27422763 PMC4983520

[34] Griffin ML McDermott KA McHugh RK Fitzmaurice GM Jamison RN Weiss RD. Longitudinal association between pain severity and subsequent opioid use in prescription opioid dependent patients with chronic pain. Drug Alcohol Depend. (2016) 163:216–21. 10.1016/j.drugalcdep.2016.04.023 27161860 PMC4880512

[35] Jha S Thakar R. Female sexual dysfunction. Eur J Obstet Gynecol Reprod Biol. (2010) 153:117–23. 10.1016/j.ejogrb.2010.06.010 20678854

[36] Stringer JD. Gender and sexual health: sexual dysfunction. FP Essent. (2016) 449:18–26. 27731968

[37] Chen L Shi GR Huang DD Li Y Ma CC Shi M. Male sexual dysfunction: a review of literature on its pathological mechanisms, potential risk factors, and herbal drug intervention. Biomed Pharmacother. (2019) 112:108585. 10.1016/j.biopha.2019.01.046 30798136

[38] McCabe MP Sharlip ID Lewis R Atalla E Balon R Fisher AD. Risk factors for sexual dysfunction among women and men: a consensus statement from the fourth international consultation on sexual medicine 2015. J Sex Med. (2016) 13:153–67. 10.1016/j.jsxm.2015.12.015 26953830

[39] McCool-Myers M Theurich M Zuelke A Knuettel H Apfelbacher C. Predictors of female sexual dysfunction: a systematic review and qualitative analysis through gender inequality paradigms. BMC Womens Health. (2018) 18:108. 10.1186/s12905-018-0602-4 29929499 PMC6013982

[40] Rees J Abrahams M Doble A Cooper A Prostatitis Expert Reference Group (PERG). Diagnosis and treatment of chronic bacterial prostatitis and chronic prostatitis/chronic pelvic pain syndrome: a consensus guideline. BJU Int. (2015) 116:509–25. 10.1111/bju.13101 25711488 PMC5008168

[41] Guan Y Yu G Wang G Bai Z. The negative effect of urologic chronic pelvic pain syndrome on female sexual function: a systematic review and meta-analysis. Int Urogynecol J. (2019) 30:1807–16. 10.1007/s00192-019-03984-z 31227839

